# Engineered nanofiber scaffolds prime chromatin reorganization to sensitize cells for long-term and low-dose chemical hazard detection

**DOI:** 10.3389/fphar.2025.1636594

**Published:** 2025-08-25

**Authors:** Yupeng Zhang, Hang Zhao, Li Jiang, Qi Zhang, Tingbao Cao, Zesheng Wang, Yang Song, Kunpeng Qu

**Affiliations:** ^1^ General Surgery Department Three, Gansu Province Central Hospital, Lanzhou, China; ^2^ Institute of Biomedical Engineering, West China School of Basic Medical Sciences & Forensic Medicine, Sichuan University, Chengdu, China; ^3^ Department of General Dentistry, State Key Laboratory of Oral Diseases, National Clinical Research Center for Oral Diseases, West China Hospital of Stomatology, Sichuan University, Chengdu, China

**Keywords:** low dose, chemical exposure, toxicology, cell sensitivity, nanofibrous scaffold, screen platform

## Abstract

Fast and early detection of low-dose chemical toxicity is a critical unmet need in toxicology and human health, as conventional 2D culture models often fail to capture subtle cellular responses induced by sub-toxic exposures. Here, we present a bioengineered three-dimensional (3D) electrospun nanofibrous scaffold composed of polycaprolactone that enhances chromatin accessibility and primes fibroblasts for improved sensitivity to low-dose chemical stimuli in a short period. The scaffold mimics the extracellular matrix, providing topographical cues that reduce cytoskeletal tension and promote nuclear deformation, thereby increasing chromatin openness. Chromatin compaction indices and accessibility assays confirmed significantly more relaxed chromatin in cells cultured on the scaffold compared to those on glass slides. Mechanistic investigations revealed that this chromatin priming effect was mediated by reduced F-actin polymerization and increased nuclear height. To evaluate functional consequences, fibroblasts were challenged with 0.1% paraformaldehyde (PFA), a commonly encountered chemical with known long-term health risks. While cells on 2D substrates showed no significant response, those on the 3D scaffold exhibited early decreases in viability and elevated ROS levels. Prolonged low-dose PFA exposure further confirmed that scaffold-cultured cells could detect cytotoxicity several days earlier than conventional controls. To facilitate clinical translation, we developed a 96-well-compatible platform by plasma-bonding scaffold-coated PDMS sheets with a custom 3D-printed well plate. Optimization of electrospinning time and cell seeding density identified conditions that maximized sensitivity and reproducibility. Then a low-dose ethanol model was developed to conclude that low-dose ethanol can affect cell viability. Together, these findings support a mechanistic model in which increased chromatin accessibility elevates the basal cellular state, expanding the “sensitive window” for detecting physical and chemical insults. This study establishes a robust and scalable platform for fast and early-phase chemical risk screening and offers a novel strategy for modulating cellular responsiveness via mechano-epigenetic regulation. The platform is broadly applicable in toxicology, pharmacology, and environmental health, offering a significant advancement in cell-based biosensing and precision diagnostics.

## 1 Introduction

Increasing evidence underscores the critical health risks associated with chronic, low-dose exposure to environmental chemicals, particularly during vulnerable developmental stages such as embryogenesis, infancy, and adolescence. In contrast to the conventional toxicological paradigm, presumes monotonic dose–response relationships and well-defined safety thresholds (Kumar et al., 2020). Growing evidence demonstrates that numerous compounds, including endocrine-disrupting chemicals (EDCs) and persistent organic pollutants (POPs), exhibit non-monotonic dose–response behavior. Such compounds can induce profound physiological effects at nanomolar to picomolar concentrations, often through mechanisms involving hormone mimicry, receptor interference, and epigenetic modifications (Gaillard et al., 2025). Prolonged low-dose exposure, even at environmentally relevant levels, can lead to cumulative biological burden, disrupting endocrine, neurodevelopmental, immune, and metabolic homeostasis. Notably, these effects are frequently latent, with clinical manifestations emerging only after long latency periods (Guéguen et al., 2019; Siama et al., 2019). The complexity of chemical mixtures further compounds these risks, such as the “mixture effect” or “cocktail effect” describes interactions that are not captured by assessments of individual agents. This presents a critical challenge for current regulatory frameworks, which often fail to account for the dynamics of low-dose, long-term, and combined exposures (Delfosse et al., 2021; Bloch et al., 2023; Docea et al., 2021).

To more accurately characterise the subtle yet consequential impacts of chronic chemical exposure on human health. Integration of advanced *in vitro* systems such as organ-on-chip platforms or high-resolution omics technologies is necessary to address these limitations (Akarapipad et al., 2021; Goralski et al., 2023; Ko et al., 2019). Due to the inherent genomic stability and environmental adaptability of somatic cells, the deleterious effects of low-dose chemical exposure often remain latent over extended periods before becoming clinically apparent. This latency poses a significant challenge for early diagnosis and intervention, as the resulting chronic damage is frequently irreversible and can culminate in severe health outcomes such as hematological malignancies (e.g., leukemia), neurodevelopmental disorders, and congenital malformations (Kang et al., 2021; Van Maele-Fabry et al., 2019; Bemanalizadeh et al., 2022; Spinder et al., 2019). Current toxicological assays, which primarily rely on acute or high-dose exposure models, are often insufficiently sensitive to capture the subtle molecular perturbations elicited by prolonged low-dose exposure (Li and Xia, 2019; Pognan et al., 2023). These limitations underscore an urgent need for innovative cellular models and analytical platforms that can amplify or sensitise cellular responses to minimal chemical insults. One of the central bottlenecks at the cellular level is the limited responsiveness of somatic cells to low-dose stimuli, which masks early-stage toxicological effects and compromises the accuracy of hazard identification. This is further complicated by the complex, non-linear dose–response relationships characteristic of many environmental toxicants, where biological effects may occur at doses previously considered safe (Lauterstein et al., 2020). Ultimately, enhancing the sensitivity and fidelity of *in vitro* systems to detect low-dose chemical toxicity is a critical step toward more predictive, human-relevant toxicological evaluations. This will facilitate timely risk assessment and contribute to the formulation of safer environmental and pharmaceutical policies.

In this study, a fast and early-stage platform was developed to detect low-dose chemical exposures. Previous studies have proved that increased chromatin accessibility can enhance cellular sensitivity to environmental stimuli (Borah et al., 2024). This finding suggests a promising strategy: by increasing the epigenetic accessibility of somatic cells, specifically through open the chromatin,it may be possible to increase their responsiveness to low-dose chemical exposures. Electrospinning is a cost-effective and scalable technology for fabricating nanofibrous scaffolds with high surface area and tunable properties. Its simple setup, compatibility with diverse polymers, and ability to produce large quantities make it highly suitable for industrial applications in tissue engineering, filtration, and beyond (Borah et al., 2024). In this study, we fabricated polycaprolactone (PCL) nanofiber scaffolds using electrospinning technology. As a classical biocompatible matrix material, the scaffold provides a three-dimensional (3D) microenvironment conducive to cellular growth. This 3D architecture can enhance cellular proliferation and promote chromatin accessibility by disrupting cytoskeletal tension and offering increased nuclear space as well. Accompanying the global increase in chromatin accessibility, cells exhibited significantly heightened sensitivity to low-dose stimuli, including chemotherapeutic agents and ultraviolet (UV) radiation. Notably, when cells were exposed to 0.1% paraformaldehyde, cells cultured on PCL scaffolds demonstrated a marked decline in viability, indicating clear responsiveness to the chemical stress. In contrast, cells maintained under conventional 2D culture conditions showed notable resistance to the same treatment, suggesting reduced sensitivity under planar culture constraints. To enable translational application, we further developed and optimized a scalable, high-throughput platform for assessing low-dose chemical toxicity, tailored to meet clinical screening demands. In sum, this work provides a novel strategy and experimental platform for the early evaluation of risks associated with low-dose chemical exposure, addressing an unmet need in preventive toxicology and environmental health.

## 2 Materials and methods

### 2.1 Electrospun scaffolds prepare

PCL nanofibers were prepared by electrospinning. PCL solution was prepared by dissolving 1 g of PCL (90 kDa, Sigma, United States) in 10 mL of hexafluoroisopropanol (HFIP, Sigma, United States) to form a 10% (w/v) solution. Electrospinning was performed in a fume hood using an open-cage target to collect fibers. The solution is then loaded into a syringe equipped with a blunt-tip metallic needle and mounted on a syringe pump set to a flow rate of 0.5–1.0 mL/h. Electrospinning is performed by applying a high voltage (12–18 kV) between the needle and a flat collector placed 12–20 cm away, allowing fibre deposition over 60 min to achieve optimal scaffold thickness. Scaffolds are sterilized prior to use by UV exposure or ethanol treatment.

### 2.2 Cell culture

Fibroblasts were isolated from the ear tissues of adult (4-week-old) C57BL/6 mice, this study has been approved by the Ethical Committee of Gansu Province Central Hospital (2023-GSFY-63). Cells were expanded in fibroblast medium consisting of DMEM (Gibco, 11,965), 10% fetal bovine serum (FBS; Gibco, 26140079), and 1% penicillin/streptomycin (Gibco, 15140122). Passage-2 cells were used for all experiments.

### 2.3 AFM measurement of cell mechanical property

To determine the elastic modulus of scaffold, mechanical measurements of single cells were performed by using atomic force microscopy (AFM) (JPK Nanowizard 4a) with tipless cantilevers (NPO-10, Bruker Corp., United States) with a 50 μm silicon beads, a highly sensitive cantilever k = 0.06 N/m, and sample Poisson’s ratio of 0.5. The force-distance curves were recorded, and the elastic modulus of cells was calculated by NanoScope Analysis using the Hertz model.

### 2.4 Cell viability assays

1 × 104 fibroblasts were seeded on the glass slide and scaffold for 24 h in a 6 well plate. Cell viability was assayed using the MTS (Promega) according to the manufacturer’s protocol. Cells were incubated with the MTS Reagent for 1 h. Absorbance was measured by a plate reader (Infinite 200PRO) at excitation/emission = 560/590 nm. Results were normalized to control (i.e., cell passing through >200 μm channels) samples.

### 2.5 DNA damage assay

DNA damage assays were performed using the HCS DNA Damage Kit (Invitrogen, H10292) according to the manufacturer’s protocol. Cells were fixed with 4% paraformaldehyde solution for 15 min at room temperature and permeabilized by 0.25% Triton^®^ X-100 in PBS for another 15 min at room temperature. Cells were washed 3 times with PBS and incubated in 1% bovine serum albumin (BSA) solution for 1 h, followed by pH2AX antibody (1:1,000) for 1 h at room temperature and then Alexa Fluor^®^ 555 goat anti-mouse IgG (H + L) secondary (1:5,000) with Hoechst 33,342 (1:6,000) for another 1 h at room temperature after removing the antibody. Epifluorescence images were collected using a Zeiss Axio Observer Z1 inverted fluorescence microscope and analyzed using ImageJ. Results were normalized to control samples.

### 2.6 Chromatin accessibility assay

Chromatin Accessibility Assay Kit (Abcam) was employed to detect the chromatin accessibility of cells which seed on the glass slide and scaffold. Simply, 24 h after cells seeded on the glass slide and scaffold, then cells were collected by Trypsin (Gibco). Following the protocol of the kit, and result were readout via PCR.

### 2.7 Immunofluorescence staining and microscopy

Samples collected for immunofluorescence staining at the indicated time points were washed once with PBS and fixed in 4% paraformaldehyde for 15 min. Samples were washed three times with PBS for 5 min each and permeabilized using 0.5% Triton X-100 for 10 min. After three subsequent PBS washes, samples were blocked with 5% normal donkey serum (NDS; Jackson Immunoresearch, 017000121) in PBS for 1 h. Samples were incubated with primary antibodies in antibody dilution buffer (1% normal donkey serum (NDS) + 0.1% Triton X-100 in PBS) for either 1 h or overnight at 4°C followed by three PBS washes and a 1-h incubation with Alexa Fluor^®^ 488- and/or Alexa Fluor^®^ 546-conjugated secondary antibodies (Molecular Probes). Nuclei were stained with DAPI in PBS for 10 min. Epifluorescence images were collected using a Zeiss Axio Observer Z1 inverted fluorescence microscope and analyzed using ImageJ. Confocal images were collected using a Leica SP8-STED/FLIM/FCS Confocal and analyzed using ImageJ. Chromatin compaction was quantified by calculating the ratio of integrated DAPI (4′,6-diamidino-2-phenylindole) fluorescence intensity to nuclear volume for each cell.

### 2.8 ELISA assay

Both Global DNA Methylation assay and ROS assay were employed ELISA assay (Abcam, ab233486 and ab186027). Samples were added to pre-coated 96-well plates provided in the kits. The plates were incubated with specific capture antibodies or binding ligands at room temperature for 4 h to allow target–receptor interaction. Following incubation, the wells were washed to remove unbound materials, and the corresponding HRP-conjugated detection antibodies were added. After a secondary incubation and final wash, the substrate solution was introduced, and the enzymatic reaction was allowed to proceed for the recommended time (30 min) in the dark. Signal intensities were measured using a microplate reader for colorimetric assays or appropriate excitation/emission wavelengths for fluorometric assays.

## 3 Result

### 3.1 Nanofibrous scaffold optimization to provide a 3D microenvironment for cell culture

**FIGURE 1:**
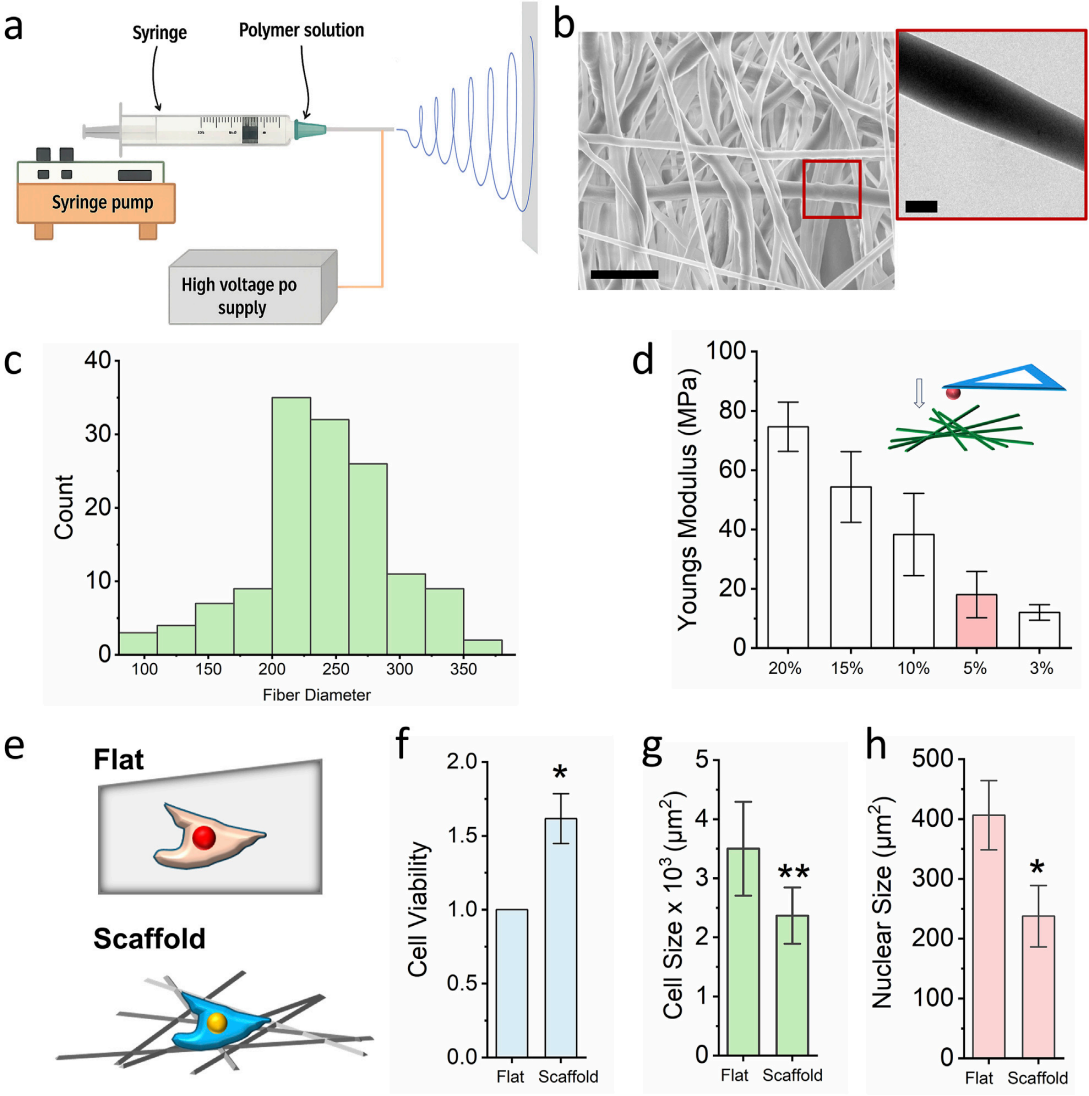
Nanofibrous scaffold preparation and optimization for cell culture. **(a)** Schematic illustrating the design of the electrospinning technology. **(b)** SEM and TEM images of the PCL nanofibrous scaffold, the scale bar for SEM = 1000 nm and he scale bar for TEM = 200 nm. **(c)** ImageJ quantifies the fiber diameter (n = 100). **(d)** AFM detects the local stiffness of the PCL scaffold with different PCL concentration. Bar graph shows mean ± SD (n = 10). **(e)** Schematic illustrating the cells seeded on the flat surface and fibrous scaffold, cells seeded on the flat surface act as control. **(f)** MTS assay detects the cell viability of cells which seed on the glass slide and 3D scaffold. Bar graph shows mean ± SD (n = 10, *p < 0.05). **(g,h)** Cell size and nuclear size of fibroblast seeded on the glass slide and nanofibrous scaffold. Bar graph shows mean ± SD (n = 50, *p < 0.05, **p < 0.01). Statistical significance was determined by a two-tailed, unpaired t-test.

To prepare a multiscale microenvironment conducive to cell culture and to modulate chromatin accessibility, electrospinning technology was employed in this study (Figure 1a). Electrospinning is a robust and scalable technique that utilizes a high-voltage electric field to produce continuous micro/nanoscale fibers from a polymer solution. As illustrated in the schematic, the polymer solution is dispensed via a syringe pump and extruded through a metallic needle. The applied electric field induces the formation of a charged polymer jet, which undergoes elongation and whipping instabilities before being deposited onto a grounded collector (flat or rotating) resulting in a nonwoven fibrous mat. These electrospun scaffolds exhibit high porosity and architectural features that closely resemble the native extracellular matrix (ECM), making them well-suited for applications in tissue engineering, regenerative medicine, and drug delivery (Dong et al., 2021).

In this study, polycaprolactone (PCL; 90 kDa) was dissolved in hexafluoroisopropanol (HFIP) to prepare the electrospinning solution. As shown in Figure 1b, scanning electron microscopy (SEM) revealed that the spinning PCL fibers ranged from 150 nm to 800 nm in diameter, with pore sizes between 0.7 μm^2^ and 19 μm^2^. Transmission electron microscopy (TEM) further confirmed the homogeneous distribution of PCL within individual fibres, supporting the scaffold’s structural integrity and suitability for three-dimensional (3D) cell culture. To optimize the local stiffness of the scaffold for chromatin regulation, PCL solutions of varying concentrations (3%–20% w/v) were electrospun and characterized. Atomic force microscopy (AFM) equipped with 50 μm spheroid tips was used to quantify the local elastic modulus. Previous studies have demonstrated that a local stiffness of approximately 20 kPa significantly enhances chromatin accessibility and facilitates phenotypic transitions (Song et al., 2021). In our experiments, a 5% PCL solution electrospun for 60 min produced scaffolds with a local stiffness of ∼20 kPa (Figure 1c). These scaffolds were selected for subsequent biological assays, with glass slides used as 2D culture control (Figure 1d).

Given that skin is one of the primary tissues exposed to environmental chemicals, fibroblast of skin is the primary cell population to interact with the chemical exposure which is also easy to collect in clinical. In this study, mouse ear-derived fibroblasts were seeded onto both the PCL scaffold and flat glass surfaces. Cell viability was assessed using the MTS [3-(4,5-dimethylthiazol-2-yl)-5-(3-carboxymeth-oxyphenyl)-2-(4-sulfophenyl)-2H-tetrazolium] assay. As shown in Figure 1e, fibroblasts cultured on the electrospun scaffold exhibited significantly enhanced viability compared to those on the 2D glass slide. Notably, both cellular and nuclear sizes were reduced in the scaffold group relative to the control group (Figures 1f-h), suggesting that the 3D fibrous microenvironment influences not only proliferation but also cellular morphology. These findings indicate that electrospun PCL scaffolds effectively recapitulate aspects of the native ECM and can prime cellular responses relevant to chromatin accessibility and chemical sensitivity.

### 3.2 Nanofibrous scaffold enhances chromatin accessibility via F-actin depolymerization

**FIGURE 2:**
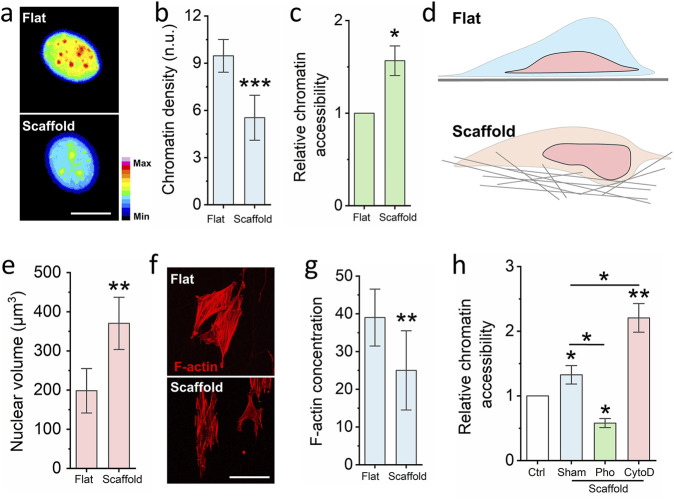
Nanofibrous scaffold increases cell chromatin accessibility. **(a)** Representative images of chromatin density on cells seed on the glass slide and nanofibrous scaffold. Scale bar = 10 μm. **(b)** DAPI staining quantifies the chromatin density of cells seed on the glass slide and nanofibrous scaffold. Bar graph shows mean ± SD (n = 50, ***p < 0.001). **(c)** Chromatin Accessibility Kit detects the chromatin accessibility of cells seed on the glass slide and nanofibrous scaffold. Bar graph shows mean ± SD (n = 3,*p < 0.05). **(d)** Schematic illustrating the cells seeded on the fibrous scaffold have higher cell volume via increased cell height. **(e)** DAPI staining quantifies the nuclear volume. Bar graph shows mean ± SD (n = 50, ***p < 0.001). **(f)** Representative images of F-actin of cells seed on the glass slide and nanofibrous scaffold. Scale bar = 100 μm. **(g)** Quantification the F-actin of cells seed on the glass slide and scaffold. Bar graph shows mean ± SD (n = 50, **p < 0.01). **(h)** Quantification chromatin accessibility of cells which are seed on the scaffold and pre-treated by the F-actin polymerization inhibitor and depolymerization inhibitor, cells seed on the glass slide act as the control. Bar graph shows mean ± SD (n = 3,*p < 0.05, **p < 0.01). Significance was determined by a one-way ANOVA and Tukey’s multiple comparison test, and a two-tailed unpaired t-test.

To investigate whether the 3D fibrous scaffold modulates chromatin compaction, we evaluated the degree of chromatin condensation in cell nuclei cultured on different substrates. Chromatin compaction was quantified by calculating the ratio of integrated DAPI (4′,6-diamidino-2-phenylindole) fluorescence intensity to nuclear volume for each cell, as previously described (Luciano et al., 2021). This metric, referred to as the chromatin compaction index, inversely correlates with chromatin accessibility—a lower value indicates a less compacted, more accessible chromatin state. Our analysis revealed that fibroblasts cultured on the electrospun scaffold exhibited significantly reduced chromatin compaction compared to those on glass slides (Figure 2a). To further validate this observation, we performed a chromatin accessibility assay. As shown in Figure 2b, fibroblasts grown on the scaffold demonstrated markedly higher chromatin accessibility than those cultured on the flat glass substrate. Interestingly, this occurred despite the reduced nuclear size observed in scaffold-cultured cells. We hypothesized that the 3D architecture of the scaffold, characterized by pore sizes ranging from 0.7 to 19 μm^2^, enables partial cellular embedding within the fibrous network, thereby facilitating vertical expansion of the nucleus (Figure 2c). Consistent with this hypothesis, nuclear volume measurements revealed a significant increase in nuclear dimensions in cells cultured on the scaffold compared to the glass surface (Figure 2d). This result further proved that increased nuclear volume can enhance the chromatin accessibility, which is same as previous reported (Wu et al., 2025).

Previous studies have established that reduced actin cytoskeletal tension can promote chromatin accessibility and drive phenotypic transitions (Soto et al., 2023). To determine whether cytoskeletal remodeling underlies the observed increase in chromatin accessibility on the scaffold, we assessed F-actin organization via Phalloidin-RFP staining. As shown in Figure 2e, cells on the scaffold exhibited markedly reduced F-actin polymerization relative to those on glass. To further interrogate this mechanism, fibroblasts were pre-treated with either Cytochalasin D (an actin polymerization inhibitor) or Phalloidin (an F-actin stabilizer) prior to seeding onto the scaffold. After 24 h of culture, chromatin accessibility was reassessed. As shown in Figures 2f-h, treatment with Phalloidin significantly reduced chromatin accessibility, whereas Cytochalasin D led to a pronounced increase. In sum, these findings demonstrate that electrospun nanofibrous scaffolds disrupt cytoskeletal organization, leading to nuclear reshaping and enhanced chromatin accessibility.

### 3.3 Nanofibrous scaffold enhances cell sensitivity to low dose environmental damage

**FIGURE 3:**
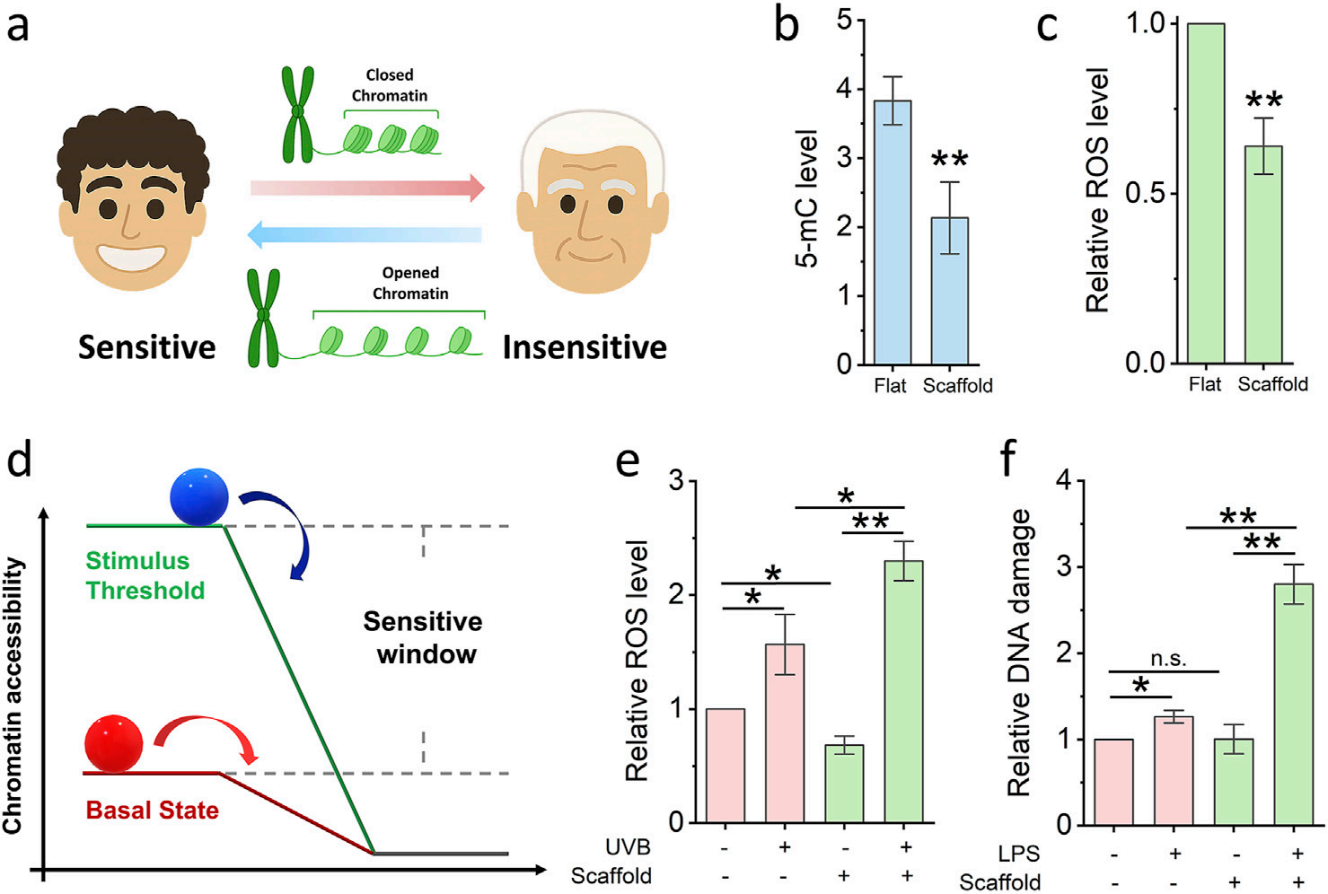
Nanofibrous scaffold increases cell sensitivity. **(a)** Schematic illustrating the relationship between cells sensitivity and chromatin accessibility. **(b,c)** ELISA quantifies the 5-mC and ROS level of cells seed on the glass slide and nanofibrous scaffold. Bar graph shows mean ± SD (n = 3, **p < 0.01). **(d)** Schematic illustrating the model of cell sensitive window. **(e)** ELISA quantifies the ROS level of cells seed on the glass slide and scaffold, then damaged by low-dose UVB. Bar graph shows mean ± SD (n = 3,*p < 0.05,**p < 0.01). **(f)** DNA damage quantified after cells seed on the glass slide and scaffold, then damaged by low-dose LPS. Bar graph shows mean ± SD (n = 3,*p < 0.05,**p < 0.01). Significance was determined by a one-way ANOVA and Tukey’s multiple comparison test, and a two-tailed unpaired t-test.

Chromatin accessibility is known to influence cellular sensitivity to environmental stimuli; increased chromatin accessibility has been shown to enhance cellular responsiveness (Figure 3a). To determine whether electrospun scaffolds can enhance cellular sensitisation and expand the range of cellular response (i.e., the “sensitive window”), fibroblasts were cultured on scaffolds for 24 h, after which levels of global DNA methylation and reactive oxygen species (ROS) were quantified via ELISA. As shown in Figures 3b,c, the level of 5-methylcytosine (5-mC) was significantly reduced in scaffold-cultured cells compared to cells on glass slides. Concurrently, ROS levels were also lower in the scaffold group. These findings suggest that the scaffold-induced increase in chromatin accessibility not only promotes a more open epigenetic state but also reduces cellular stress, thereby enhancing the cells’ sensitivity to environmental cues.

To conceptualize the observed differences in cellular responsiveness, we propose a mechanistic model (Figure 3d) wherein chromatin accessibility acts as a key determinant of the cell’s basal energetic or regulatory “state platform.” In this model, cells with low chromatin accessibility (represented by the red ball on a lower platform) are positioned at a reduced baseline, limiting their capacity to detect and respond to subtle environmental stimuli. Their response is only triggered once external stimuli surpass a relatively high activation threshold, resulting in a narrow “sensitive window.” By contrast, cells with increased chromatin accessibility (depicted by the blue ball on a higher platform) occupy an elevated regulatory state. This heightened state lowers the threshold required for stimulus perception, thereby expanding the sensitive window the range within which cells can detect and respond to perturbations. The vertical distance between the two platforms in the diagram illustrates the difference in basal readiness, while the horizontal component reflects the dynamic range of response. As a result, cells with greater chromatin accessibility are primed for more rapid and robust transcriptional responses to both chemical (e.g., lipopolysaccharide) and physical (e.g., UVB radiation) stimuli, as demonstrated in our experimental assays. This model supports the notion that chromatin architecture is not merely a passive indicator of transcriptional potential, but an active regulator of how cells interpret and respond to environmental inputs. It further suggests that bioengineered platforms, such as 3D nanofibrous scaffolds can be strategically designed to tune epigenetic states and thereby modulate cell sensitivity for diagnostic, therapeutic, or screening applications.

To experimentally validate this model, we subjected fibroblasts to low-dose physical and chemical challenges. UVB radiation (2 kJ/m^2^ for 10 min) was used to induce oxidative stress. As shown in Figure 3e, ROS levels increased significantly more in scaffold-cultured cells than in glass-cultured cells following UVB exposure, indicating heightened sensitivity. Additionally, lipopolysaccharide (LPS), a well-characterised inducer of DNA damage, was applied at a low concentration (0.5 μM) for 2 h. As shown in Figure 3f, LPS treatment resulted in a greater degree of DNA damage in cells cultured on scaffolds compared to those on glass, further supporting the notion of scaffold-enhanced cellular sensitivity. In summary, the 3D fibrous scaffold microenvironment promotes chromatin relaxation and reduces basal stress, thereby priming cells to be more responsive to both physical and chemical environmental stimuli.

### 3.4 Low dose paraformaldehyde reduce cell viability on nanofibrous scaffold

**FIGURE 4:**
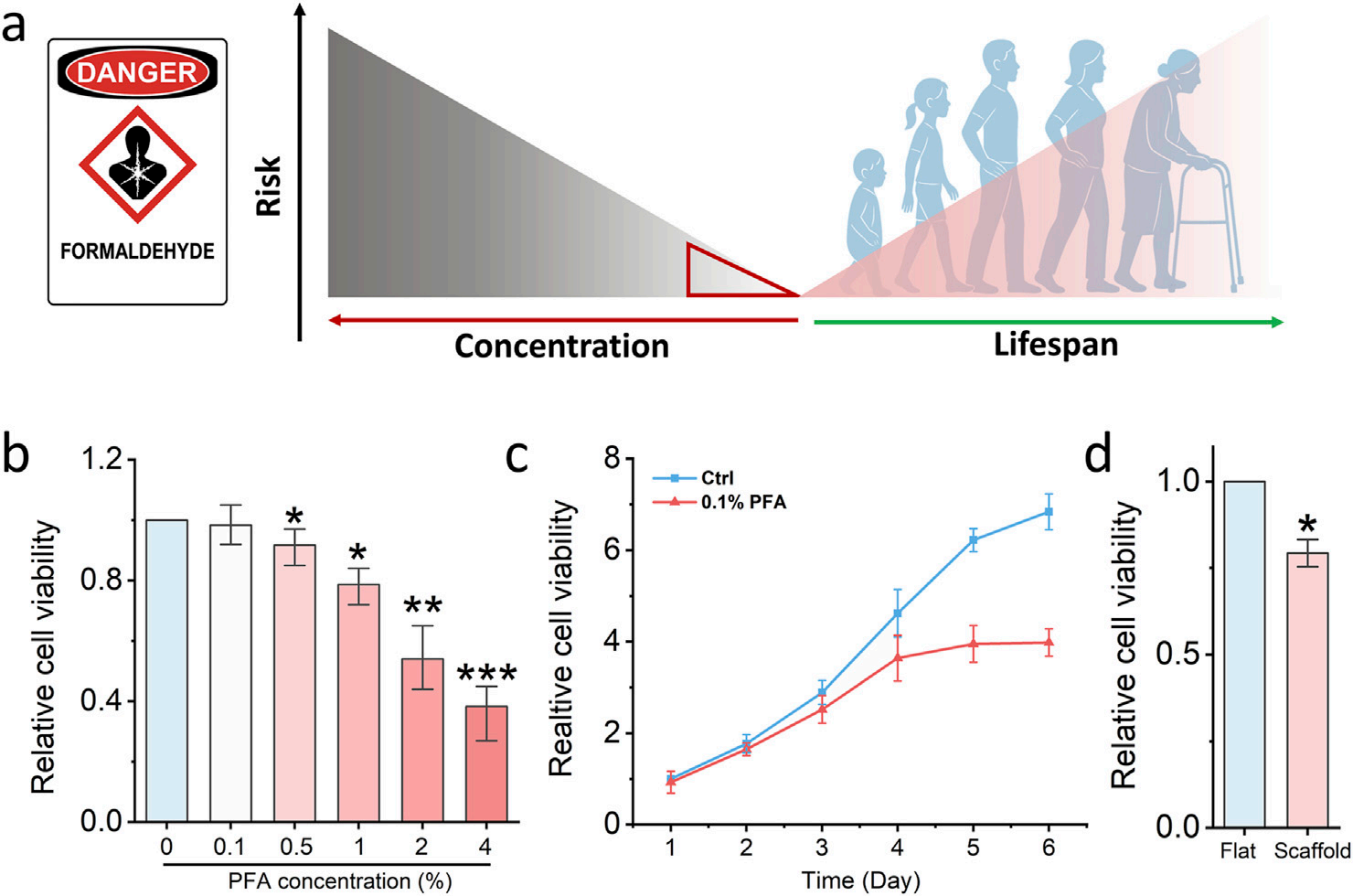
Nanofibrous scaffold detects low-dose formaldehyde damage. **(a)** Schematic illustrating the relationship between low-dose formaldehyde damage to the human lifespan. **(b)** MTS assay detects the cell viability of fibroblasts which are treated by different concentrations PFA. Bar graph shows mean ± SD (n = 3,*p < 0.05,**p < 0.01, ***p < 0.001). **(c)** MTS assay detects the cell viability of fibroblasts which are treated by low-dose PFA for 6 days. Bar graph shows mean ± SD (n = 3). **(d)** MTS assay detects the cell viability of fibroblasts which seed on the glass slide and fibrous scaffold then treated by low-dose PFA. Bar graph shows mean ± SD (n = 3,*p < 0.05). Significance was determined by a one-way ANOVA and Tukey’s multiple comparison test, and a two-tailed unpaired t-test.

To assess whether electrospun nanofibrous scaffolds enhance fibroblast sensitivity to low-dose chemical exposure and thereby enable early detection of potential cytotoxic risk, we selected paraformaldehyde (PFA) as a model compound. Formaldehyde is a well-characterized chemical fixative known to induce DNA damage, impair cell viability, and contribute to the development of serious diseases such as nasopharyngeal carcinoma and leukemia (La Torre et al., 2023). While high concentrations of PFA are acutely toxic, increasing epidemiological and experimental evidence suggests that chronic exposure to low doses, such as those encountered in occupational or polluted environments, can also elicit adverse biological effects over time, including carcinogenesis (Figure 4a).

In our study, fibroblasts were first seeded in standard 96-well plates and exposed to a range of PFA concentrations to establish a toxicity threshold. As shown in Figure 4b, 0.1% PFA did not significantly affect cell viability under conventional 2D culture conditions, indicating that such a concentration falls below the acute toxicity threshold. To further investigate the long-term effects of low-dose PFA exposure, fibroblasts were cultured in 96-well plates and treated continuously with 0.1% PFA for 6 days. As shown in Figure 4c, a notable decline in cell viability was observed after 4 days of treatment, supporting the notion that even low concentrations of PFA can exert cumulative cytotoxic effects over time. However, we hypothesised that cells cultured on a 3D scaffold primed by increased chromatin accessibility might exhibit heightened sensitivity and thus be capable of detecting sub-toxic exposures at earlier time points. To test this, fibroblasts were seeded on the electrospun PCL scaffold for 24 h and subsequently treated with 0.1% PFA for another 24 h. Remarkably, a significant reduction in cell viability was observed in scaffold-cultured fibroblasts compared to those grown on glass (Figure 4d). This suggests that the scaffold microenvironment sensitizes cells to otherwise undetectable levels of chemical stress.

Together, these findings demonstrate that electrospun scaffolds enhance the cellular detection of low-dose toxicants and enable earlier identification of chemical-induced cytotoxicity. This platform offers a promising approach for early risk screening in environmental toxicology and public health surveillance.

### 3.5 Low-dose chemical exposure risk detection platform preparation and optimization

**FIGURE 5:**
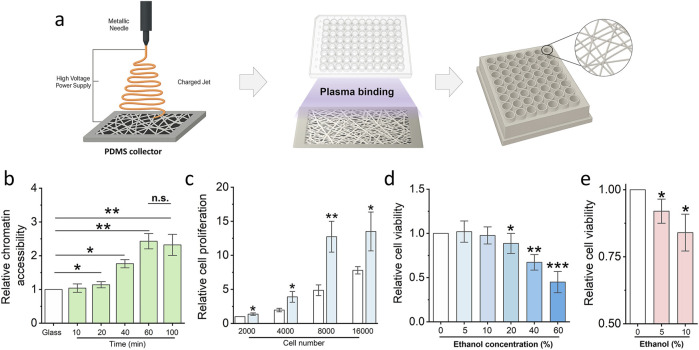
Low-dose chemical exposure detection platform generation and optimization. **(a)** Schematic illustrating the protocol of the low-dose chemical exposure detection platform generation. **(b)** Cell Chromatin accessibility assay utilized to optimize the electronspun time of the platform generation. Bar graph shows mean ± SD (n = 3, *p < 0.05, **p < 0.01). **(c)** MTS assay detects the cell proliferation of fibroblasts to optimize the cell number for the low dose exposure risk assay. Bar graph shows mean ± SD (n = 3, *p < 0.05, **p < 0.01). **(d)** MTS assay detects the cell proliferation of fibroblasts which are treated via different concentrations ethanol. Bar graph shows mean ± SD (n = 3, *p < 0.05, **p < 0.01, ***p < 0.001). **(e)** Fast check platform detect the potential risk of low-dose ethanol via MTS assay. (n = 3, *p < 0.05). Significance was determined by a one-way ANOVA and Tukey’s multiple comparison test, and a two-tailed unpaired t-test.

To facilitate the clinical translation of scaffold-induced cell sensitization for early detection of low-dose chemical toxicity, we developed a modular, high-throughput 3D scaffold-based platform compatible with standard 96-well plate formats. As illustrated in Figure 5a, nanofibrous scaffolds were first fabricated on flat poly-dimethylsiloxane (PDMS) collectors via electrospinning. Subsequently, a custom-designed 3D-printed 96-well cover plate was aligned and bonded to the scaffold-coated collector surface using a 1-min plasma treatment, ensuring firm adhesion without compromising fiber integrity. The resulting integrated device is amenable to parallelized analysis and enables systematic evaluation of chemical toxicants at scale, offering a promising solution for screening environmental or pharmaceutical compounds under physiologically relevant 3D conditions.

To optimize platform performance for maximal sensitivity, we systematically evaluated two critical parameters: electrospinning duration and initial cell seeding density. As shown in Figure 5b, a 60-minute electrospinning period yielded scaffolds with optimal thickness and fibre density to significantly enhance chromatin accessibility compared to shorter durations. No further improvement was observed with extended spinning beyond 60 min, indicating a plateau in mechano-regulatory effect. Concurrently, cell proliferation analysis revealed that a seeding density of 8,000 fibroblasts per well provided the highest sensitivity to stimuli while maintaining robust viability (Figure 5c). This condition was therefore selected for subsequent low-dose chemical exposure assays. Low-dose ethanol was utilised to further evaluate the platform’s capability. The results demonstrated that 10% ethanol does not impair cell viability after 24 hours of exposure; however, prolonged exposure to the same concentration leads to a significant decline in cell viability (Figures 5d,e).

In summary, we have established and optimized a scalable, clinically oriented platform that integrates electrospun nanofibrous scaffolds with standardized well-plate geometry. This system enables early-stage detection of low-dose chemical toxicity by leveraging the epigenetically primed, sensitized state of fibroblasts cultured in 3D. With its potential for high-throughput screening and compatibility with existing laboratory infrastructure, this platform provides a powerful tool for toxicological evaluation in both research and translational settings.

## 4 Discussion

In this study, we present a novel 3D nanofibrous scaffold platform capable of priming chromatin accessibility and enhancing cellular sensitivity to low-dose chemical exposure. Through electrospinning polycaprolactone (PCL) fibers onto flat collectors and integrating them into a modular well-plate format, we successfully recapitulated a physiologically relevant microenvironment that promotes nuclear remodeling and chromatin decondensation. Our data demonstrates that fibroblasts cultured on this scaffold exhibit reduced chromatin compaction, increased nuclear height, and heightened sensitivity to chemical and physical stimuli. These effects are mechanistically linked to decreased cytoskeletal tension and increased nuclear pliability—conditions that favor transcriptional activation and rapid epigenetic response. This chromatin “priming” effect significantly lowers the detection threshold for toxicants such as paraformaldehyde, enabling earlier and more sensitive evaluation of cytotoxic risk compared to conventional 2D cultures. To achieve the same purpose, different platform was developed to enhance the chromatin accessibility for cell microenvironment detection, such as soft hydrogel and patterned substrates (Heo et al., 2023; Luo et al., 2022). In this study, we did not set the hydrogel with same local stiffness as a control, since the local stiffness of electrospun scaffold we used is around 20 MPa, this is difficult to prepare a hydrogel with such a high local stiffness via AFM measurement.

The clinical importance of detecting low-dose and early-phase toxicological insults cannot be overstated (Serras et al., 2021). Traditional toxicology models, which often rely on high-dose exposure paradigms, fail to capture subtle molecular perturbations that occur at physiologically relevant exposure levels (Serras et al., 2021). Our findings show that cells grown on the electrospun scaffold respond robustly to sub-toxic concentrations of 0.1% PFA, exhibiting decreased viability and elevated stress markers well before these changes manifest in standard 2D cultures. Additionally, long-term low-dose exposure experiments reveal cumulative cytotoxic effects consistent with epidemiological data linking chronic low-level chemical exposure to diseases such as leukemia and nasopharyngeal cancer (Rühm et al., 2022). By offering a window into this early response phase, our platform provides a unique opportunity to understand and monitor environmentally induced cellular damage at its inception.

Beyond toxicology, this platform has broad translational potential. The modular scaffold-plate system is compatible with standard 96-well formats, making it scalable for high-throughput screening and adaptable for pharmaceutical testing, environmental monitoring, and personalized medicine. By optimizing scaffold thickness and cell seeding density, we have established conditions that maximize chromatin accessibility and detection sensitivity without compromising cell viability. This approach could be extended to other cell types and stressors, including radiation, metabolic toxins, and epigenetic drugs. Ultimately, our scaffold-based system represents a next-generation diagnostic tool that bridges the gap between fundamental cell biology and real-world clinical and environmental challenges, offering a sensitive, tunable, and physiologically meaningful platform for early toxicity screening (Roffel and Hoogdalem, 2024).

## Data Availability

The original contributions presented in the study are included in the article, further inquiries can be directed to the corresponding author.
